# In Memoriam—John K. Lee, M. D.

**Published:** 1887-12

**Authors:** 


### JOHN K. LEE, M. D.

Dr. John K. Lee died very suddenly at his home, in this city, early Thursday morning, November 10th. Dr. Lee was a prominent and successful homœopathic physician. He had filled many important offices in State and county medical societies, was a member of the State Board of Charities, etc.

